# Comparative Data Are Needed for Pulmonary Artery Aneurysm Management Guidelines

**DOI:** 10.1016/j.atssr.2025.10.001

**Published:** 2025-11-04

**Authors:** AlleaBelle Bradshaw, Emmanuel Robinson, Ahmet Kilic

**Affiliations:** Division of Cardiac Surgery, Department of Surgery, Johns Hopkins University, 1800 Orleans St, Baltimore, MD 21287

To the Editor

With many uncommon diseases like pulmonary artery aneurysms (PAAs), the optimal management strategies remain uncertain. We commend Kagawa and coworkers^1^ for their case series of 11 PAAs that were treated surgically. Given that most of the existing literature on PAAs consists of isolated case reports, their series is valuable. Using their experience and prior studies,^2^ they derive an algorithm for treating PAAs at their institution.

Prior reviews have highlighted that PAAs carry heterogeneous risks, including rupture, dissection, and compression of adjacent structures.^2^ However, incidence of these complications is low, and predictors are poorly defined. Thus, caution is warranted before generalizing treatment algorithms from only descriptive data. Although Kagawa and coworkers reported 11 surgically treated PAAs, there were 218 non–surgically treated patients at their institution during the same period, and their outcomes were not analyzed. Without such comparative data, the relative risks and benefits of surgical intervention remain speculative, based on theoretical risk. There is currently no strong evidence to support a surgical indication for PAAs.

Consider patients who have undergone Ross procedures, in whom the pulmonary autograft frequently dilates over time. Surgeons often do not intervene unless there is significant aortic insufficiency. Furthermore, if operations are offered, they are usually attempting to salvage the autograft rather than because of the risk of adverse events.^3^ In other words, if pulmonary autografts in the aortic position rarely lead to complications despite exposure to high pressures, it is reasonable to expect that PAAs in the native low-pressure pulmonary circulation would be even less likely to do so. Admittedly, this expectation is based only on pathophysiology logic.

Another limitation of the series is that underlying patient diseases are unknown.^1^ One patient with pulmonary hypertension and a pulmonary artery diameter of only 42 mm underwent a double-lung transplant, whereas another with a giant PAA (109 mm) required prolonged postoperative extracorporeal membrane oxygenation support. These cases represent distinct and complex clinical scenarios, making it difficult to compare them or to derive generalizable management guidelines based on limited information about each patient.

With robust data-sharing collaborations now established between institutions in the United States, comparative data should be attainable. A multi-institutional database of patients with pulmonary artery enlargement could lead to truly informed guidelines. Until such data exist, management should emphasize individualized risk markers rather than algorithmic thresholds that may expose patients to operative risk without demonstrated benefit.

## References

[1] Kagawa H., Silvester B., Smego D. (2025). Surgical therapy for pulmonary artery aneurysm. Ann Thorac Surg Short Rep.

[2] Kreibich M., Siepe M., Kroll J. (2015). Aneurysms of the pulmonary artery. Circulation.

[3] Martin E., Mohammadi S., Jacques F. (2017). Clinical outcomes following the Ross procedure in adults: a 25-year longitudinal study. J Am Coll Cardiol.

